# Effects of Isolation Among Older Adults Due to Coronavirus Restrictions

**DOI:** 10.1093/geroni/igab046.1860

**Published:** 2021-12-17

**Authors:** Cynthia Thomas

**Affiliations:** International Network for the Prevention of Elder Abuse, Rockville, Maryland, United States

## Abstract

Semi-structured interviews were conducted with 35 residents in a Maryland condominium, four to six months after the presence of the epidemic in the US was recognized in mid-March. The objective was to determine to what extent the restrictions resulting from the presence of a new disease was affecting older adults in their daily lives, and in their plans for the future. All respondents were over the age of 60 and half were more than 80 years old. Two-thirds lived by themselves; most others lived with a husband or wife. Respondents for the most part were following guidelines to wear masks, practice social distancing and avoid close contact with persons outside their homes, including other family members. Over half had already made dramatic changes in their daily activities. Some found an opportunity to develop new skills, had connected with people from the past, or had become more introspective. Others, while exhibiting some of the same characteristics, were more focused on the restrictions they faced, and were more aware than ever of the limited amount of time left in their lives. Differences between respondents in the emphasis of their perspectives are explored, by age, gender, and other characteristics.

